# Case Report: Atypical Presentation of Visceral Leishmaniasis: Two Cases from Northwest Ethiopia

**DOI:** 10.4269/ajtmh.20-0666

**Published:** 2021-04-12

**Authors:** Rezika Mohammed, Helina Fikre, Tigist Mekonnen, Bewketu Abebe, Arega Yeshanew, Ermias Diro, Johan van Griensven

**Affiliations:** 1 Department of Internal Medicine, University of Gondar, Gondar, Ethiopia;; 2 Leishmaniasis Research and Treatment Center, University of Gondar, Gondar, Ethiopia;; 3 Department of Pathology, University of Gondar, Gondar, Ethiopia;; 4 Department of Clinical Sciences, Institute of Tropical Medicine, Antwerp, Belgium

## Abstract

Human visceral leishmaniasis (VL) is a life-threatening disease caused by protozoan parasites belonging to the *Leishmania donovani* complex. Atypical cases of leishmaniasis and HIV coinfection have been documented in case reports, mostly associated with gastrointestinal tract, kidney, and skin involvement. We report two VL cases with atypical localizations not reported from east Africa before, both diagnosed and treated at the Leishmaniasis Research and Treatment Center, Gondar, Ethiopia. The first case was an HIV-infected patient with scrotal and penile involvement. *Leishmania* parasites were detected in the spleen and the scrotum. The second case was an immunocompetent individual with esophageal, laryngeal, and pharyngeal involvement and facial lesions. *Leishmania* parasites were detected in the spleen, skin, and esophageal biopsies. Current evidence suggests atypical presentation can occur in patients irrespective of their HIV status. Therefore, we suggest a high index of suspicion for VL among clinicians working in endemic areas of Ethiopia.

## INTRODUCTION

Human visceral leishmaniasis (VL) is a life-threatening disease caused by protozoan parasites belonging to the *Leishmania donovani* complex. Visceral leishmaniasis primary infects macrophages in the deep organs such as the spleen, liver, and bone marrow, leading to the classical presentation of VL which is fever, loss of appetite, weight loss, hepatosplenomegaly, and progressive pancytopenia. Currently, east Africa carries the highest VL burden globally. Ethiopia carries the highest rate of VL/HIV coinfection, reaching between 20% and 40% in some parts of the country.^1^

Visceral leishmaniasis and HIV mutually reinforce each other. Visceral leishmaniasis increases the HIV viral load and accelerates progression to AIDS. Conversely, VL is more severe in HIV patients, with higher rates of treatment failure, death, and relapse.^2,3^ Atypical clinical presentation involving tissues outside the reticuloendothelial system such as gastrointestinal and renal systems were reported among HIV patients.^4,5^ This is thought to be related to the severe immunosuppression in VL/HIV patients and poor parasite containment leading to dissemination of the parasite to atypical sites. Atypical site involvement is also seen in patients with immunosuppressive conditions other than HIV.^6–8^

We have previously reported four atypical cases of VL in HIV patients in Ethiopia, including three with gastrointestinal involvement and one with unusual skin involvement. Another study, also from Ethiopia, reported three cases of disseminated cutaneous leishmaniasis (CL) resembling post–kala-azar dermal leishmaniasis (PKDL), caused by *L. donovani*.^9^ We now report two cases of atypical VL with localizations not reported from east Africa before.

## CASE 1

**Figure 1.**
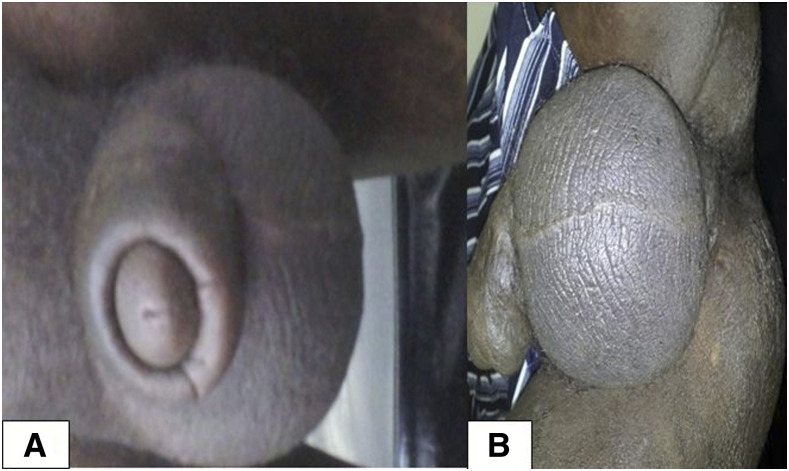
Patient with visceral leishmaniasis and HIV coinfection with (**A**) swelling of the penile shaft and (**B**) diffuse scrotal swelling with a nodular lesion (arrow) at the base of the scrotum. This figure appears in color at www.ajtmh.org.

The first case relates to a 40-year-old male patient on first-line antiretroviral therapy for 9 years with good adherence. He presented to the Leishmaniasis Research and Treatment Center, Gondar, Ethiopia, with high-grade intermittent fever of 1 month associated with loss of appetite and unquantified weight loss. He had a scrotal swelling since a year and had been treated for presumed lymphogranuloma venereum (LGV) 5 months earlier, and got some improvement. On physical examination, his vital signs were stable with no fever recorded. There was an enlarged spleen (seven centimeter below the left costal margin) and liver (6 cm below the right costal margin). The scrotum was diffusely swollen with a circumferential length of 23 cm × 22 cm, involving also the penile shaft. There was a small nodular lesion at the base of the scrotum (Figure 1A and B). His complete blood count test showed pancytopenia. From a spleen aspiration Leishman–Donovan bodies were identified, with a parasite grade of +6.^10^ Skin slit from the base of the scrotum also showed Leishman–Donovan bodies (parasite load was not graded). At admission, his CD4 count was 54 cells/µL, despite having an undetectable viral load. Ultrasound examination of the scrotum showed thickening of the scrotal wall with a small amount of fluid around both testes with echo-debris. Both testes were of normal size, shape, and echotexture.

With the diagnosis of primary VL involving the scrotum and penis, he was treated with AmBisome and miltefosine combination treatment for 2 months and discharged with negative tissue aspiration results in the spleen and scrotum. By the end of the treatment, the scrotal swelling subsided, hemogram improved, and CD4 count increased to 140 cells/µL levels.

## CASE 2

**Figure 2.**
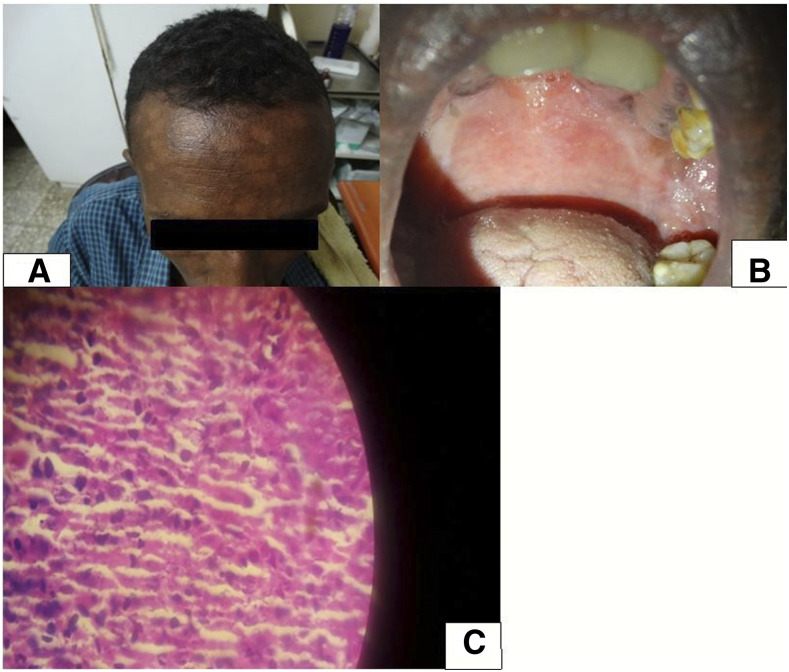
Patient with visceral leishmaniasis showing (**A**) hypopigmented facial lesions, (**B**) palatal erythema and swelling; (**C**) microscopic image of Leishman–Donovan body from esophageal biopsy. This figure appears in color at www.ajtmh.org.

A 28-year-old man presented with hypopigmented lesions on the face existing since 2 years. The lesion became scaly after a year (Figure 2A). He also reported difficulty swallowing, odynophagia, and feeling a mass during swallowing, which progressively worsened. Two weeks before the presentation, he started to have gum bleeding, high-grade intermittent fever, loss of appetite, and unquantified significant weight loss.

On physical examination, his vital signs were stable. The soft palate and pharynx were diffusely swollen and erythematous (Figure 2B). There was bilateral submandibular lymphadenopathy (1 cm × 1 cm). There was an enlarged spleen (6 cm below the left costal margin) and liver (2 cm below the right costal margin). There were multiple small, macular, patchy hypopigmented lesions on the face (Figure 2A). The complete blood count and renal and liver function tests were in the normal range. The recombinant K39 antigen (rK39) rapid diagnostic test was positive. The HIV test was negative. Amastigotes were detected by microscopy from the spleen aspirate, skin slit, and endoscopic biopsy from the esophagus (Figure 2C). Treatment was started with sodium stibogluconate and paromomycin combination therapy for 30 days. As the palatal lesion and dysphagia had not resolved by day 30, sodium stibogluconate was continued for another month. At end of the 2-month treatment, the patient was asymptomatic and no parasites were detected in the spleen. The patient came for 3- and 6-month posttreatment follow-ups, and no reappearance of the skin and/or palatal lesions were observed.

## DISCUSSION

We report the first case of VL with scrotal involvement and the first case with esophageal, laryngeal, and palatal involvement from east Africa. There have been reports of genital involvement in VL from other parts of the world. Similar to our case, a case was reported in 1960 from the United Kingdom, initially diagnosed with syphilis but with VL subsequently confirmed.^11^ In our patient, the partial response to LGV treatment, having a treatment history for other sexually transmitted disease (STD) (urethral discharge) in 2008, and being coinfected with HIV, may suggest that he may have had concomitant LGV attributable for the scrotal swelling. This case may alert clinicians working in VL-endemic areas to think of leishmaniasis in patients diagnosed with an STD and investigate more to confirm the diagnosis. More research on *Leishmania* infections mimicking STDs and the prevalence of coinfections of *Leishmania* and STDs is warranted.

The second case presented with skin lesions, which were followed by mucosal involvement (esophageal, laryngeal, and palatal) and later on visceral involvement, with the disease progressing over a 2-year period. Unlike most VL cases, the reason this patient progressed over such a long period might be explained by the fact that he was not known to have an immunocompromising condition. There have been reports of esophageal and laryngeal leishmaniasis in different regions of the world.^12–14^ Cutaneous lesions may occur before or after the development of VL, including macules, papules, nodules, and ulcers. In our patient, the skin lesions were macular (Figure 2A) and preceded the systemic VL manifestations. There are reports of visceralization by species usually causing CL, and vice versa, there are reports of skin manifestations from species causing VL.^1,9^ In Ethiopia, CL is mainly caused by *L aethiopica*. In our patient, the fact that the patient traveled in the VL-endemic area and lives in an area where there is no CL and the positive rK39 result make it likely that he had VL with atypical presentation. Another mimicker to our case is PKDL, a skin lesion which mostly develops after successful treatment of VL, but in 15–20% of the cases, PKDL can happen before VL.^15,16^ This mimicry has been reported from Sudanese patients as well.^17^ In our patient, the lesions happened before VL, so PKDL followed by VL cannot be excluded. One limitation of our report is that species identification could not be carried out in our setting. The two cases, in addition to the previous reports, provide evidence to fill the gap in the leishmaniasis guidelines of developing countries like Ethiopia. The cases demonstrate for clinicians working in endemic areas to have a high index of suspicion of disseminated VL in patients presenting with atypical presentations, including in HIV-negative patients.
